# Supervised Machine Learning for Classification of the Electrophysiological Effects of Chronotropic Drugs on Human Induced Pluripotent Stem Cell-Derived Cardiomyocytes

**DOI:** 10.1371/journal.pone.0144572

**Published:** 2015-12-22

**Authors:** Christopher Heylman, Rupsa Datta, Agua Sobrino, Steven George, Enrico Gratton

**Affiliations:** 1 Department of Biomedical Engineering, University of California, Irvine, Irvine, CA, United States of America; 2 Laboratory of Fluorescence Dynamics, University of California, Irvine, Irvine, CA, United States of America; 3 Edwards Lifesciences Center for Advanced Cardiovascular Technology, University of California, Irvine, Irvine, CA, United States of America; 4 Department of Molecular Biology and Biochemistry, University of California, Irvine, Irvine, CA, United States of America; 5 Department of Biomedical Engineering, Washington University in St. Louis. St. Louis, MO, United States of America; University of California, Berkeley, UNITED STATES

## Abstract

Supervised machine learning can be used to predict which drugs human cardiomyocytes have been exposed to. Using electrophysiological data collected from human cardiomyocytes with known exposure to different drugs, a supervised machine learning algorithm can be trained to recognize and classify cells that have been exposed to an unknown drug. Furthermore, the learning algorithm provides information on the relative contribution of each data parameter to the overall classification. Probabilities and confidence in the accuracy of each classification may also be determined by the algorithm. In this study, the electrophysiological effects of β–adrenergic drugs, propranolol and isoproterenol, on cardiomyocytes derived from human induced pluripotent stem cells (hiPS-CM) were assessed. The electrophysiological data were collected using high temporal resolution 2-photon microscopy of voltage sensitive dyes as a reporter of membrane voltage. The results demonstrate the ability of our algorithm to accurately assess, classify, and predict hiPS-CM membrane depolarization following exposure to chronotropic drugs.

## Introduction

Machine learning is generally defined as the process of training an algorithm (i.e. learning) to make predictions or decisions based on data. Supervised learning is a subtype of machine learning in which a set of training data with known classifications or outcomes is used to train the algorithm by building a statistical model that fits the training data. The model can then be applied to unknown data to predict the classification or outcome of each datum. In the case of predicting cardiac side effects of drug treatments, electrophysiological data from actively beating cardiomyocytes acquired after exposure to drugs with known chronotropic effects may be used to train a classification algorithm based on defined parameters of the depolarization waveform. The trained algorithm can then be applied to data acquired from cardiomyocytes exposed to drugs whose effects are unknown to screen for electrophysiological effects and similarities to existing drugs. Furthermore, this highly quantitative approach can be used to assess the relative contribution of each waveform parameter to the algorithm’s classification decision. Confidence in predictions and similarities to existing drugs can also be measured. Supervised learning techniques have come to the fore in recent years for a number of biological and potentially clinical applications including ECG signal quality, systemic vascular resistance, and decoding functional magnetic resonance imaging (fMRI) of brain states [1–3]. The pressing need for automated high-throughput analysis of electrophysiology at the cell membrane level is apparent, but remains unsolved. Thus, a supervised learning algorithm is a potential method to fill this need, especially when combined with recent advances in electrophysiological data acquisition. Juhola et al. have previously used a machine learning approach to classify calcium cycling anomalies in human cardiomyocytes [4], however, to our knowledge, the study presented here demonstrates the first application of machine learning to interpret the membrane voltage of human cardiomyocytes following drug treatment.

Many drugs showing promise in preclinical trials fail during clinical development due to the emergence of cardiac side effects [5–10]. Hence, there exists a great need to develop novel in vitro platforms that more accurately mimic the biology of human cardiac cells, and thus provide a reliable and accurate model for high-throughput drug screening. The emergence of human induced pluripotent stem (hiPS) cell technology has created a new source of human cardiomyocytes (hiPS-CM) [11,12]. Novel microscopy and analysis methods serve to accelerate development and validation of such in vitro hiPS-CM models for drug screening.

Voltage sensitive dyes (VSDs) allow non-invasive, non-destructive, and longitudinal assessment of hiPS-CM electrophysiology at the sub-cellular scale [13] with reduced toxicity. The use of VSD has a long history and their utility has been demonstrated in neuronal [14] and cardiac [15–18] cells and tissues. Numerous families and formats of VSDs exist that allow wide use across microscopy platforms and cell types [13]. This study uses di-4-ANE(F)PPTEA, a hemicyanine class dye, that embeds itself in the cellular membranes of hiPS-CM and exhibits a proportional increase in fluorescence intensity as the voltage across the membrane increases. Using laser scanning 2-photon microscopy at a single location on the cell membrane, the fluorescence intensity of the VSD can be captured as a function of time, and be used to measure hiPS-CM membrane depolarization. Subcellular resolution of transmembrane voltages during action potentials may provide additional insights into cell electrophysiology and drug responses which patch clamp and other techniques cannot achieve. Analysis of the resultant signal requires compensation for artifacts due to the physical contractile motion of spontaneously beating hiPS-CM. Methods for compensation of such artifacts will be briefly discussed in this study.

The chronotropic drugs propranolol and isoproterenol were selected to validate our machine learning algorithm. These drugs were selected due to their extensive mechanistic and functional characterization of their electrophysiological effects [19]. Briefly, propranolol and isoproterenol are known to act primarily on the β-adrenoreceptors in cardiac cells as a β-blocker and β-adrenergic agonist, respectively. β-adrenoreceptors are coupled to G_αs_-proteins, which when stimulated, lead to a cascade mediated by adenylyl cyclase formation of cyclic adenosine monophosphate (cAMP) and subsequent activation of cAMP-dependent protein kinase (PKA). Activated PKA phosphorylates L-type calcium channels increasing calcium influx during an action potential. Calcium transport from the L-type calcium channels is associated with the plateau phase of a cardiac action potential. With this a priori knowledge of the action of these drugs, modulation of calcium transport (as with β-blockers and β-adrenergic agonists) would be expected to affect the plateau height and width of the depolarization waveform. The upslope and downslope of a cardiac action potential would not be expected to be affected by β–adrenergic modulation since these metrics are primarily driven by fast sodium channels and delayed rectifier potassium channels (I_KS_, I_KR_, I_K1_), respectively.

In this study, we use 2-photon microscopy to assess voltage sensitive dye (VSD) embedded in the cell membrane of actively beating human cardiomyocytes derived from induced pluripotent stem cells (hiPS-CM). We use supervised machine learning to develop an algorithm that can accurately report the effects of the chronotropic drugs, propranolol and isoproterenol. This study demonstrates the use of supervised learning to automatically and accurately assess, classify, and predict the membrane depolarization of hiPS-CMs. Although used in conjunction with electrophysiological data acquired from 2-photon microscopy of VSD in this study, this type of learning algorithm may be applied to data from any type of cardiomyocyte electrophysiological or contractility signal (e.g. patch clamp [20], microelectrode array [21], calcium reporters [22], atomic force microscopy measurements, or scanning probe microscopy measurements [23]). The application of machine learning to the study of cardiac function has the potential to be very useful in the development of high-throughput methods for drug discovery to identify drugs that are potentially cardiotoxic.

## Methods

### Human induced pluripotent stem cell-derived cardiomyocyte (hiPS-CM) culture and differentiation

Human induced pluripotent stem cells (hiPS), (wtc11 line derived as previously reported [24] and generously provided by Dr. Bruce Conklin) were subjected to a previously reported protocol that utilizes a serum-free defined medium [25] for differentiation into cardiomyocytes-like cells. Briefly, this protocol consists of culture in Roswell Park Memorial Institute (RPMI) medium (Life Technologies, 22400–071) supplemented with B-27 without insulin (Life Technologies, A1895601). On Day 0, media is supplemented with 12 μM CHIR99021 (Selleckchem, S2924) for 24 hours, then removed. On day 3, the media is supplemented with 5 μM IWP2 (Tocris, 3533). On day 5 the IWP2 is removed and on Day 7, the media is supplemented with insulin. After Day 7, cells are fed RPMI/B-27 (+) insulin (Life Technologies, 17504–044) every 2–3 days for the duration of the experiment. Cells began spontaneously beating on approximately Day 12–15, and were stained with voltage sensitive dye (VSD) and imaged on Day 33.

### Voltage Sensitive Dye (VSD) Staining

Culture medium was replaced with fresh medium containing 1uM Di-4-ANE(F)PPTEA (purchased from Leslie Loew, University of Connecticut) and incubated for 15 min at 37°C. Cells were rinsed with RPMI/B-27 (+) insulin one time and then allowed to recover for at least 2 hours prior to imaging.

### Drug Exposure

After staining with VSD, cells were qualitatively confirmed to still be spontaneously beating before addition of drugs. Images were captured immediately prior to drug exposure. Medium was then replaced with fresh medium containing either 10^−5^ M propranolol (SIGMA, P0884) or 10^−7^ M isoproterenol (SIGMA, I6504). We determined the IC_50_ of propranolol and EC_50_ of isoproterenol relative to beat rate for these cells was on the order of 10^−6^ M and 10^−9^ M (S1 Fig). We selected concentrations larger than these to ensure an effective response. Medium without drugs was also replaced as a control. Data was collected 15 min after addition of drugs to ensure complete exposure.

### 2-photon Microscopy of VSD

All measurements were obtained on a Zeiss Laser Scanning Microscope (LSM 710) (Carl Zeiss, Jena, Germany) using a 40X water immersion objective (C-Apochromat 40X/1.20 W Korr M27). A titanium:sapphire Mai Tai laser (Spectra-Physics, Mountain View, CA) was employed to excite the VSD at 850nm. A dichroic at 760 nm was used to separate excitation from emission signal. VSD fluorescence signal was collected between 489–645 nm. Temporal VSD depolarization data was acquired in line scan mode with 128 pixels per line and a 1.58 μs pixel dwell time. 100,000 line scan repeats were acquired for each measurement. All microscope components and acquisition processes were controlled using the Zen software package (Zeiss, Jena Germany). Clusters of spontaneously beating cardiomyocytes were identified in brightfield mode. The system was then switched to the parameters specified for 2-photon microscopy, a line drawn that crossed 1–5 cell membranes, and the signal acquired. After completion of data acquisition, cells were again observed in brightfield mode to confirm that they were still spontaneously beating. All microscopy was performed on a temperature-controlled stage held at 37°C that was contained within a temperature-and gas-controlled incubator held at 37°C and 5% CO_2_.

### Data Analysis and Parameter Quantification

**Fig 1:**
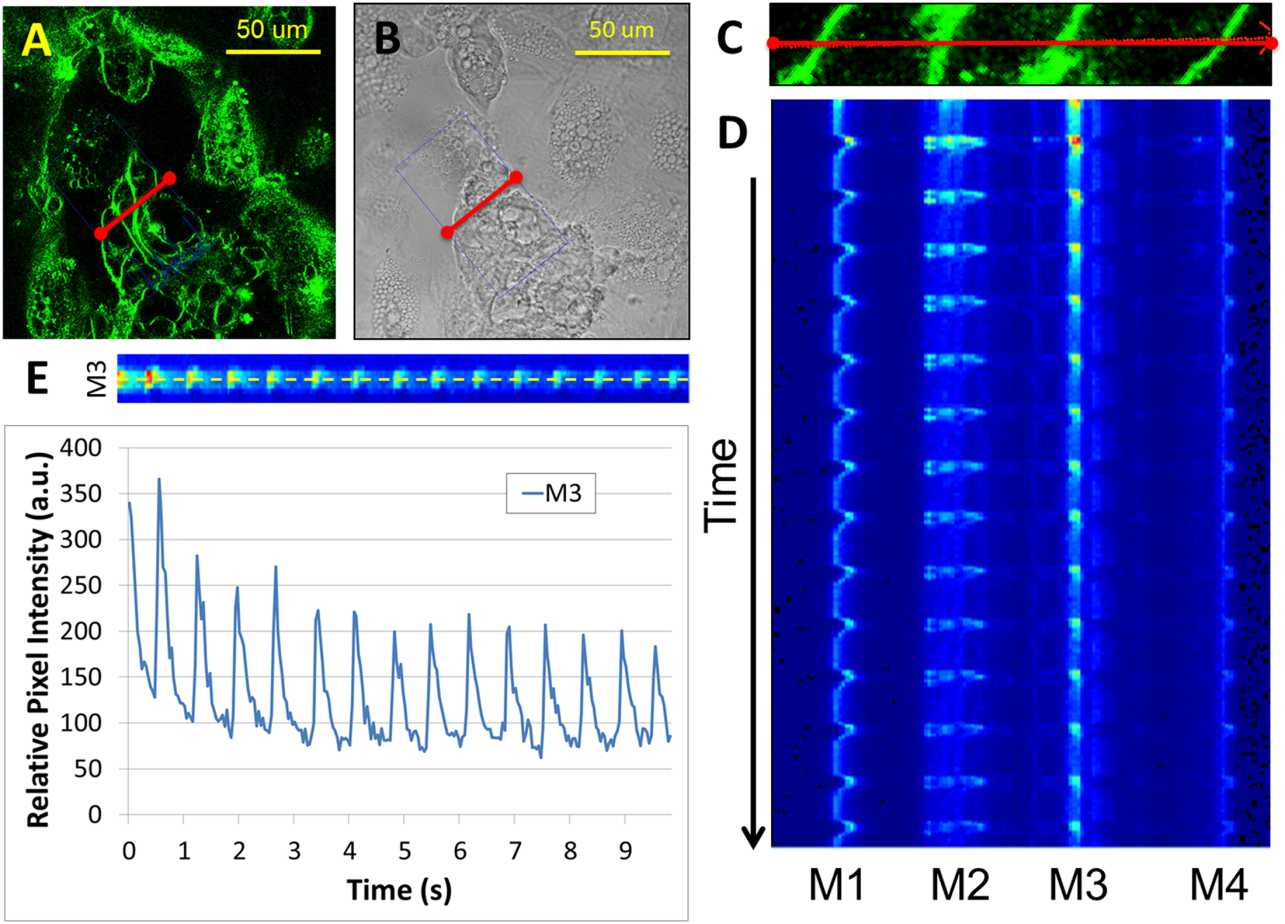
Membrane depolarization of spontaneously beating human induced pluripotent stem cell-derived cardiomyocytes (hiPS-CM). Cells were stained with voltage sensitive dye, di-4-ANE(F)PPTEA (A). Corresponding brightfield image (B). Consecutive line scans were performed using 2-photon microscopy on sets of individual cell membranes as shown in (A) and the corresponding zoom image (C). The resultant temporal sequence of line scans with heat-mapped fluorescence intensity prior to motion correction (M 1–4 specifies the individual membrane numbers) (D). Subsequent sample quantification of fluorescence intensity of a given membrane over time (M3 is the subset of the temporal line scan sequence for membrane 3) (E).

Raw fluorescence data was analyzed using the SimFCS software developed in the Laboratory of Fluorescence Dynamics (LFD, University of California, Irvine). Motion artifact resulting from the spontaneous beating of cell clusters was compensated for in data post-processing using a Gaussian tracking and correction algorithm (S2 Fig). Fluorescence intensity along each corrected cell membrane trace was extracted (Fig 1). The resultant quantification data was passed into a custom MATLAB (Mathworks, Natick, MA) script that corrected for photobleaching artifacts by fitting a second order exponential function to the data (S3 Fig and S1 Code). This fit was subtracted from the data. The resultant data was then normalized to the exponential fit (Fig 1E). The script then filtered the signal using a moving average filter with a 50 line window and detected the parameters of maximum height, upslope, downslope, peak width, and plateau height for each peak (S1 Code). The parameters were extracted by identifying the peak and trough of each depolarization waveform and then identifying the upslope, downslope, and maximum height relative to the peak/trough pair. The maximum height (h_max_) is defined as the maximum amplitude of the waveform. The upslope (m_up_) and downslope (m_down_) are defined as the slopes at 50% of the maximum height. The peak width (w) is defined as the distance from upslope to downslope at 50% of the maximum height. The plateau height (h_plateau_) is defined as the height of the waveform at the midway point of the peak width.

### Training and Validation of Classification Algorithm

The TreeBagger supervised machine learning function in MATLAB (MathWorks, 2014b, 8.4.0.118713) was used to train an algorithm to classify a single depolarization waveform based on each specific experimental condition (S1 Code). The TreeBagger algorithm utilizes random forests with bootstrap aggregation (i.e. bagging) to train the algorithm how to classify waveforms [26]. Random forests are built using the basic unit of a decision tree, a sequence of binary decisions based on model parameter values that best separate the data into their respective classification (i.e. which drug treatment they received). A random forest is assembled by constructing a large number of decision trees with a random subset of model parameters used to define each split in the tree. The mode of the classifications acquired from using all of these different decision trees in the random forest determines the ultimate classification of a given data set. This helps prevent overfitting that can occur when only one decision tree is utilized. Random selection of model parameters to define candidate splits also allows for quantification of which model features are the strongest predictors of classification. In addition, bootstrap aggregation, or bagging, is used to reduce variance and overfitting of the training data. Bagging is sampling with replacement from the training data set. If all of the trees generated in the random forest were trained using the same training data set, they would all be sensitive to the noise within that data and be correlated. By bagging and averaging the results for each tree generated, the variance is reduced and the correlation between trees is also reduced. An optimal number of trees can be determined by assessing out-of-bag error (the mean prediction error using bootstrap samples that don’t contain a given datum). We empirically determined the necessary number of decision trees to be N = 50 trees for our data set to prevent over fitting (i.e. to reach stable and minimized out-of-bag error).

This algorithm was chosen due to the type of input data (numerical parameters) and the desired learning outcome (classification of drug treatment). For these inputs and desired outputs, the TreeBagger algorithm is a robust bagging method that produces high classification accuracy. Three conditions or classifications were defined: control, propranolol, and isoproterenol. The parameters previously defined (maximum height, upslope, downslope, peak width, and plateau height) were used as input for the algorithm. To train the algorithm, the relative distribution of each treatment type was quantified (control: 145 waveforms (32% of total data set), propranolol: 100 waveforms (22% of total data set), isoproterenol: 212 waveforms (46% of total data set)). Next, 33% of the total data set, in the same proportions of treatments as the total data set (control: 45 waveforms (32% of training data set), propranolol: 34 waveforms (22% of training data set), isoproterenol: 70 waveforms (46% of training data set)) were randomly selected and used to train the algorithm using 50 different decision trees generated at random by the algorithm to classify the data. The remaining data were randomized and used as ‘unknown’ data to validate the algorithm after training. Model accuracy was quantified using the out-of-bag classification errors versus number of grown trees as a more general measure of model accuracy. The receiver operation characteristic (ROC) was used as a more specific measure of accuracy as a function of treatment. To build the ROC curves, the true positive rate (TPR) and false positive rates (FPR) were calculated based on the out-of-bag classification score, a measure of the confidence of classification as a given treatment. The TreeBagger algorithm can also quantify the relative importance of each model feature (e.g. maximum height, upslope, etc.). Using this feature, we removed the 2 most irrelevant features (using the arbitrary metric of out-of-bag feature importance <1 that qualitatively best separated the features) from the model. We then retrained and revalidated using the reduced model in order to demonstrate the effects of model simplification on classification accuracy. To determine the simplest model that still provides satisfactory classification, we continued to remove features one at a time until a significant difference in accuracy resulted.

### Statistical Analysis

Waveform parameters were quantified using waveforms from untreated samples (N = 145), waveforms treated with propranolol (N = 100), and waveforms treated with isoproterenol (N = 216). Effects of treatment were determined using Student’s t-test with α = 0.05. True positive rate/false positive rate ratios are compared graphically using the area under the curve (AUC), with AUC = 1 indicating a perfect classification algorithm with zero errors. The probability of classification of a given waveform as each treatment group was quantified for each waveform in the ‘unknown’ fraction of the data (N = 100 waveforms from untreated samples, N = 66 waveforms treated with propranolol, N = 142 waveforms treated with isoproterenol). Effects of treatment, classification, and model complexity were determined using one-way ANOVA with α = 0.05 and post-hoc Tukey’s tests were performed on relevant effects with α = 0.05.

## Results

### Drug-induced alteration of hiPS-CM membrane depolarization waveforms

**Fig 2:**
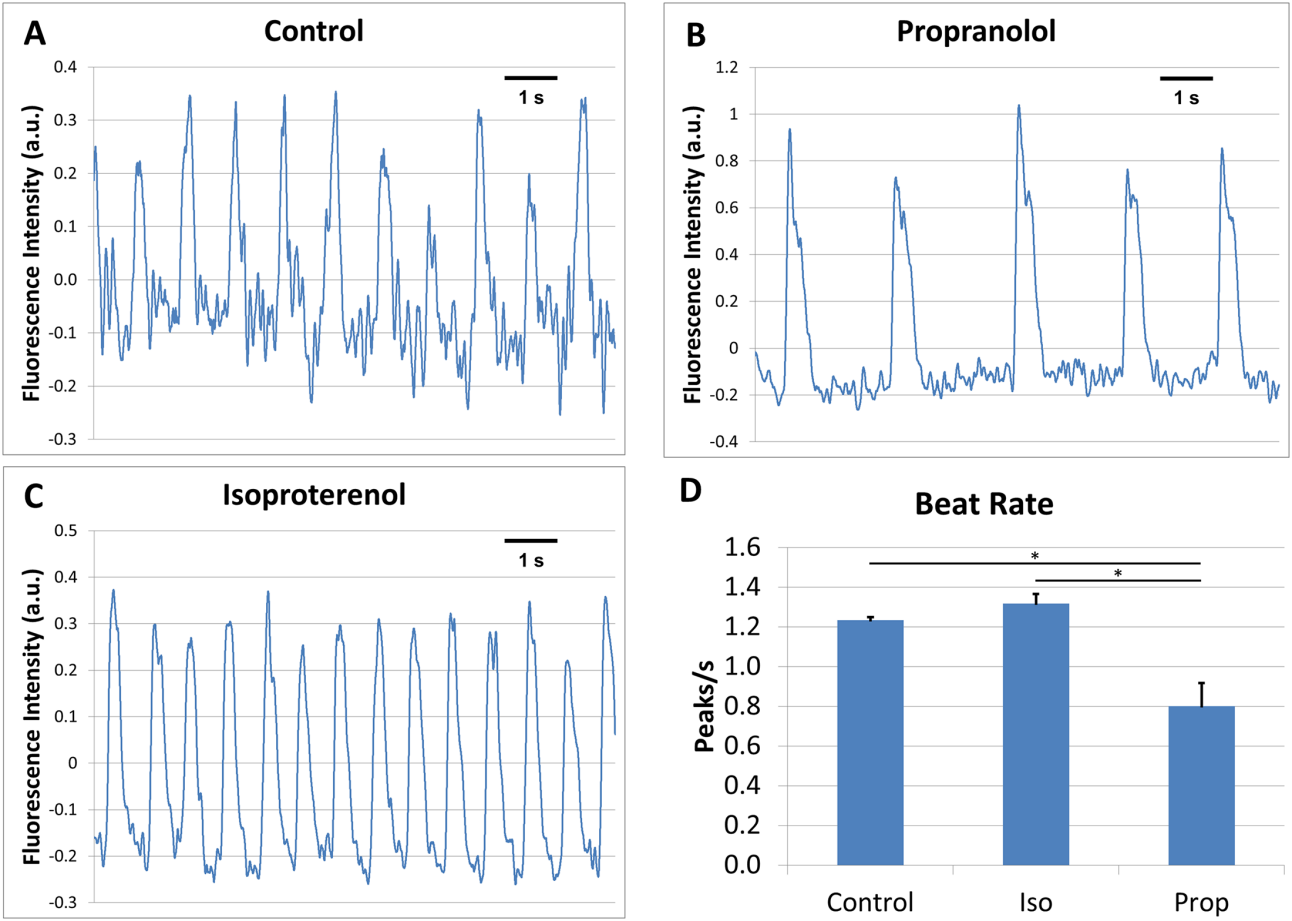
Drug induced alteration of membrane depolarization waveforms of spontaneously beating hiPS-CM. Representative traces (corrected for photobleaching) are presented for the control, N = 145 (A), propranolol-treated, N = 100 (B), and isoproterenol-treated, N = 216 (C) were administered to separate cultures. Quantification of beat rate (D). All error bars are SE. (*) indicates p<0.05 for a given comparison.

**Fig 3:**
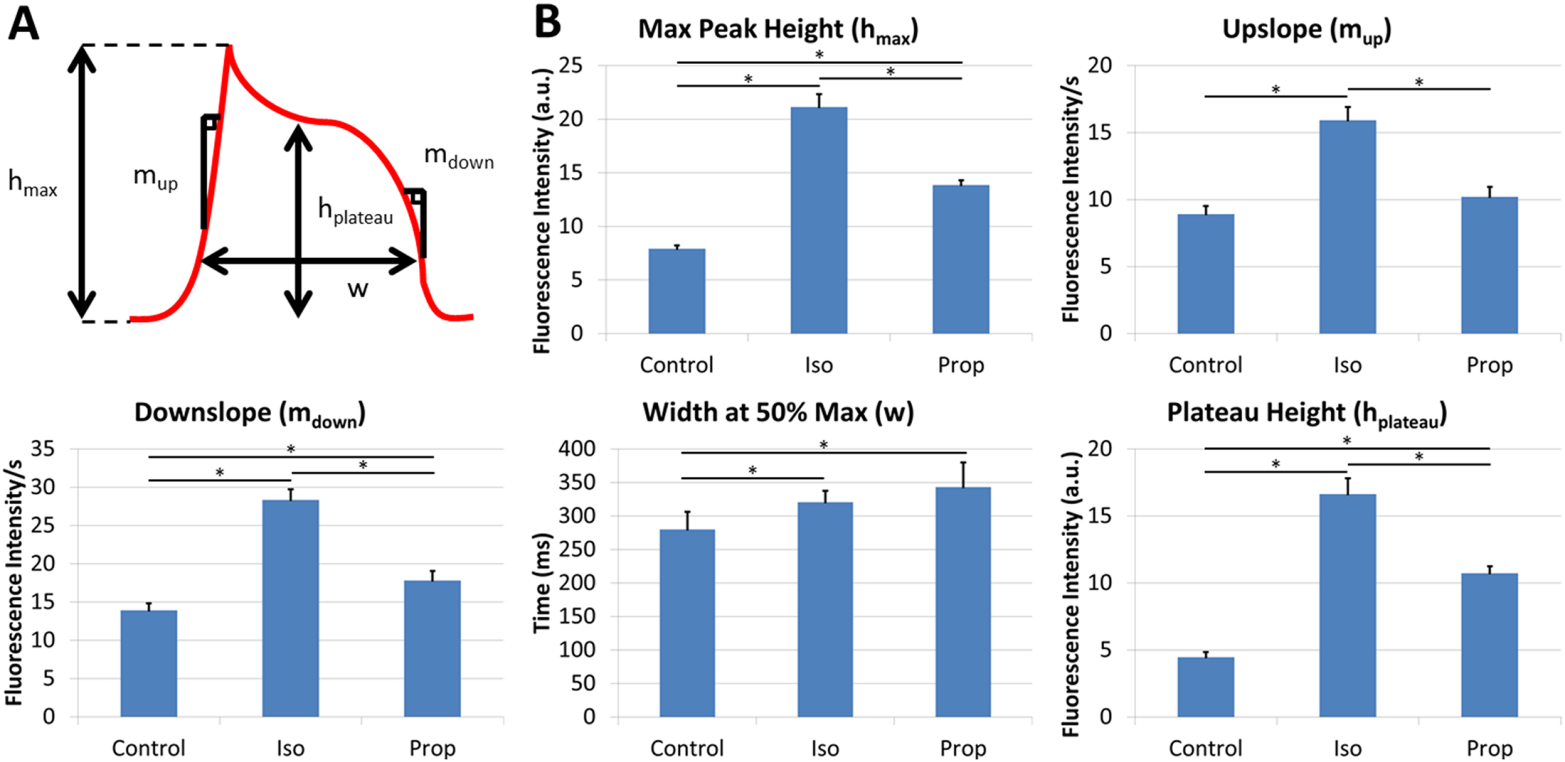
Average waveform metrics. Metric definitions: The maximum height (h_max_) is defined as the maximum amplitude of the waveform. The upslope (m_up_) and downslope (m_down_) are defined as the slopes at 50% of the maximum height. The peak width (w) is defined as the distance from upslope to downslope at 50% of the maximum height. The plateau height (h_plateau_) is defined as the height of the waveform at the midway point of the peak width (A). Average of waveform metrics (B). All error bars are SE. (*) indicates p<0.05 for a given comparison.

Data from control waveforms, propranolol exposed waveforms, and isoproterenol exposed waveforms revealed significant (p<0.05) differences across all three conditions in upslope, max height, plateau height, downslope, and peak width (Figs 2 and 3).

### Training an accurate drug treatment classification algorithm

**Fig 4:**
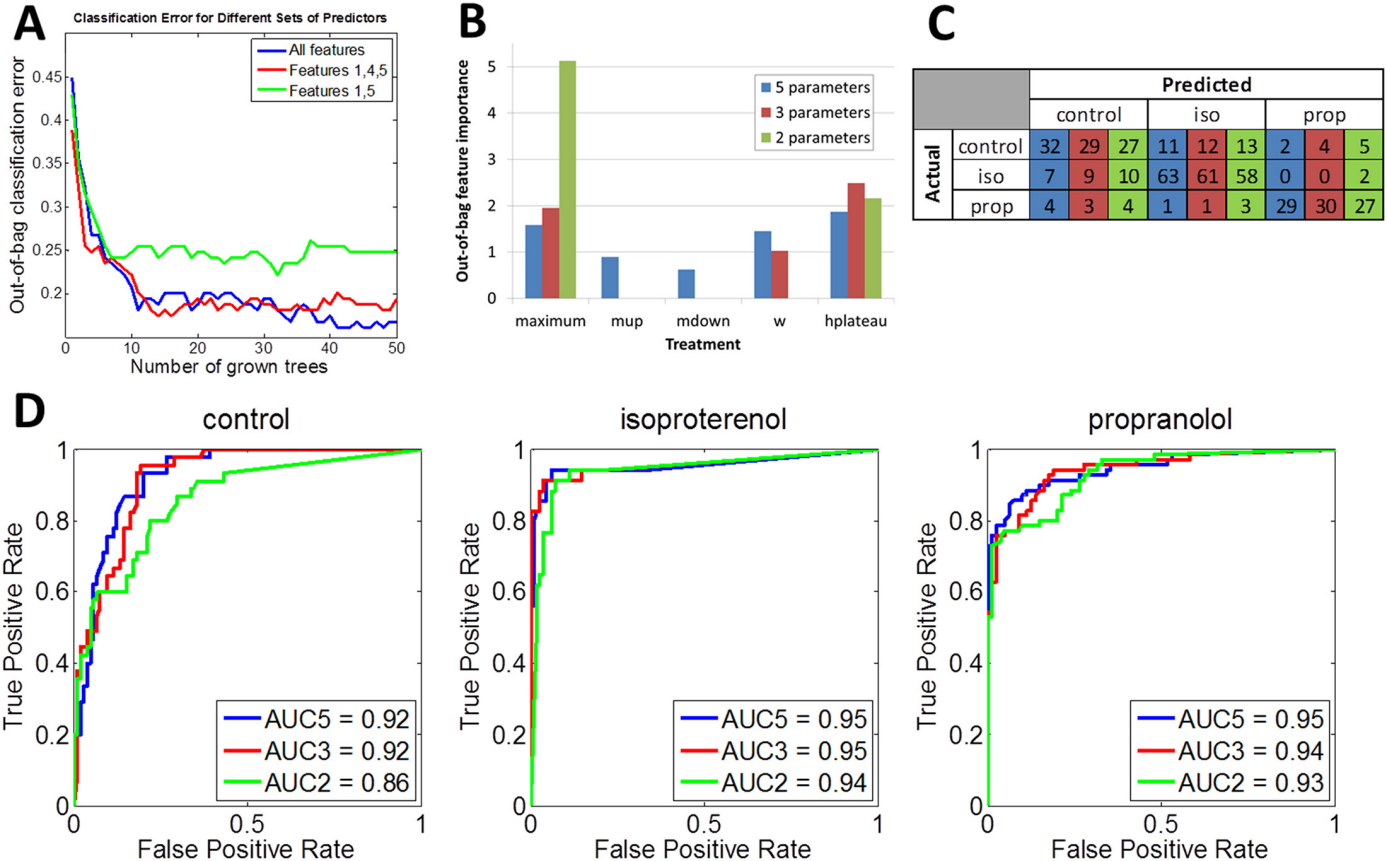
Model simplification. Metrics with the least relative importance to classification were removed sequentially. Blue = 5 parameter model, Red = 3 parameter model (upslope (m_up_) and downslope (m_down_) removed), Green = 2 parameter model (upslope (m_up_), downslope (m_down_), and width (w) removed). Relative classification errors as a function of number of grown decision trees (A). Relative importance of waveform metrics to classification determination (B). Accuracy of classification using training data set in matrix format (C) and FPR/TPR plots (D). AUC(N) = area under curve for ‘N’ parameter model.

Fig 4 reports the drug treatment classification accuracy using N = 45 control waveforms, N = 34 propranolol exposed waveforms, and N = 70 isoproterenol exposed waveforms to train the algorithm using TreeBagger. 50 decision trees were sufficient to achieve a stable out-of-bag classification error. Up to 500 trees were tested, but all data presented were obtained using 50 trees for training. 83% of individual waveforms were correctly classified during training (Fig 4C). The most common misclassification was a false positive prediction of isoproterenol treatment for a waveform that was actually a control (7% of waveforms). The area under the curve (AUC) of true positive/false positive rate plots is indicative of the overall accuracy of the model, with an AUC = 1 indicating zero errors in classification. AUC for control was 0.92, for propranolol was 0.95, and for isoproterenol was 0.95, indicating excellent prediction accuracy for each condition (Fig 4D).

### Trained algorithms reliably classify individual waveforms according to drug treatment

**Fig 5:**
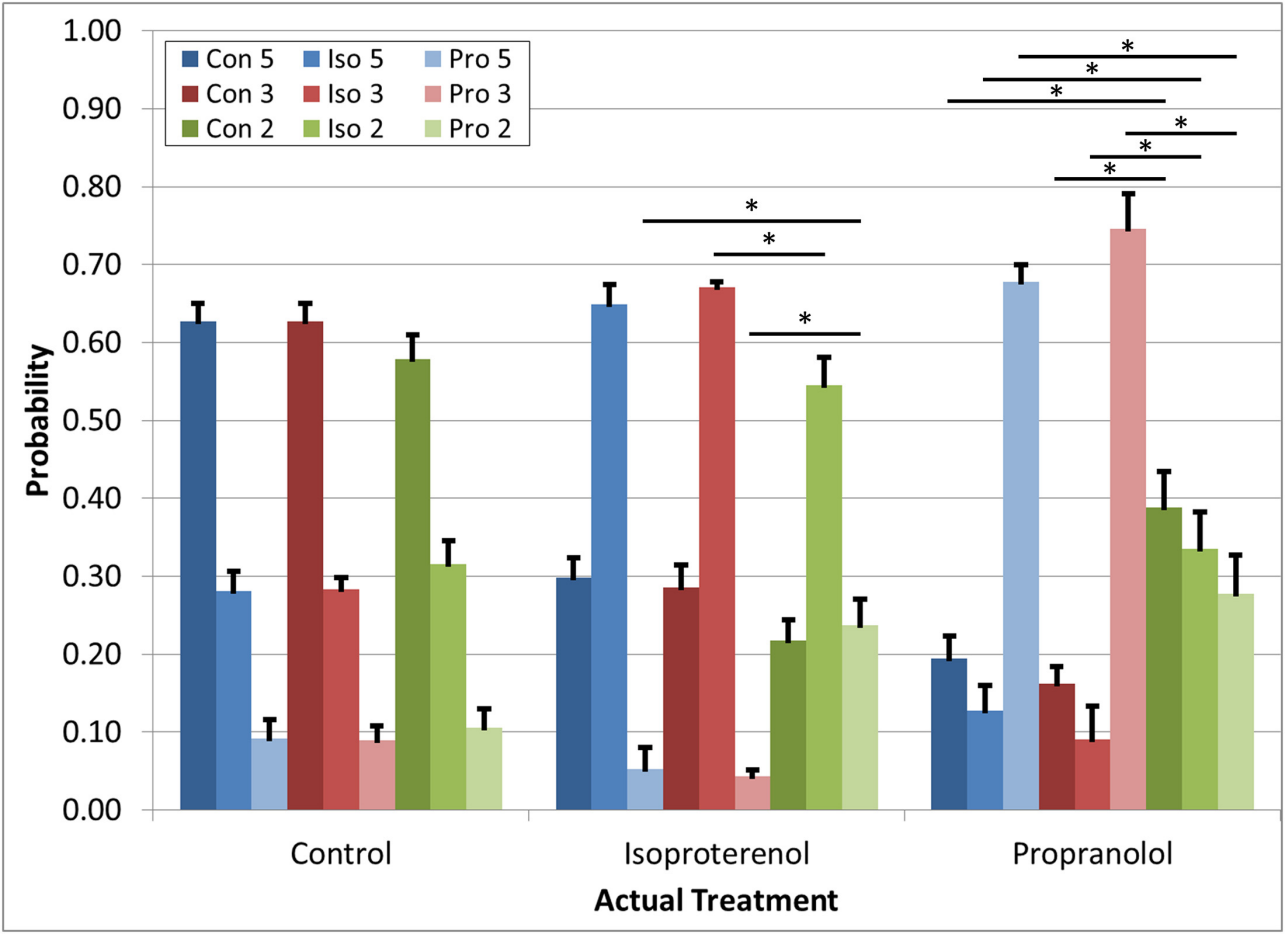
Randomized blinded “unknown” waveform data classification. Performed using reduced models (blue shades = 5 parameter model, red shades = 3 parameter model, green shades = 2 parameter model). Data are mean probability of correct classification of a single waveform as each treatment +/- SE (Con = control, Iso = isoproterenol, Pro = propranolol). The x-axis defines the actual treatment of a given waveform. (*) indicates p<0.05 for a given comparison between models (number of parameters) within a predicted treatment group.

Using the algorithm trained with a randomly selected 33% of the data collected (N = 149 waveforms), the remaining 66% (N = 308) of the data were used to characterize the accuracy of (or validate) the algorithm. The validation data was comprised of the same percentage of each treatment as the training data (control: 100 waveforms (31% of total data set), propranolol: 66 waveforms (22% of total data set), isoproterenol: 142 waveforms (46% of total data set)). 70% of individual waveforms were correctly classified (Table 1). The classification of a single waveform is a discrete selection based simply on the algorithm’s calculation of the highest probability of correct classification. With three possible classifications (control, propranolol, isoproterenol), it is therefore possible for the algorithm to select a classification with only slightly higher than a 33.3% probability of correct classification. In our algorithm, the average probability of a given waveform being classified correctly was greater than 60% for any given treatment. The average probability of incorrect classification of a given waveform was less than 30% for any given treatment (Fig 5). Practically, as a tool for predicting drug exposure, classification will be performed on an entire recording of a membrane rather than individual waveforms. When predictions of individual waveforms were aggregated over the length of an entire recording of a membrane (1 min long containing 50–100 beat waveforms), the treatment was correctly identified 100% of the time.

**Table 1:** Model Accuracy as a Function of the Number of Model Parameters.

Total Accuracy (312 Peaks)	Parameters		
	5	3	2
# Correctly Identified Waveforms	218	234	154
% Correctly Identified Waveforms	70%	75%	49%

### The sensitivity and relative importance of input parameters to waveform classification can be used to simplify and improve the model

After analysis of the relative importance of each parameter, the 2 least important parameters (upslope (m_up_) and plateau height (h_plateau_), Fig 4B), were removed from the model. The 3 parameter model accuracy was assessed and compared to the accuracy obtained using 5 parameters (Fig 4). The model performed similarly well with only 3 parameters and increased the correctly classified individual waveforms to 75% (Table 1) with significant improvements in isoproterenol and propranolol-treated classification accuracy (Table 2). The TPR/FPR plots changed shape, but the AUC for each changed no more than 0.58% for any treatment (Fig 4D). There were no significant effects between the 3- and 5-parameter models in the probability of accurate classification (Fig 5). We then removed the next least important parameter (width (w)) and again assessed model accuracy. In this case, the OOB classification error significantly increased from less than 0.2 to approximately 0.25 (Fig 4A), and the TPR/FPR plots had significantly lower AUC values (6.9% decrease for control, 2.3% decrease for isoproterenol, and 0.7% decrease for propranolol) (Fig 4D). The number of correctly classified individual waveforms dropped drastically to 49% (Table 1). The ability to correctly identify propranolol waveforms was particularly affected and reduced to 27% (Table 2). We deemed this decrease in accuracy unacceptable and performed the remaining analyses using the optimal 3 parameter simplified model.

**Table 2:** Model Accuracy by Treatment as a Function of the Number of Model Parameters.

% Correctly Identified Waveforms by Treatment	Parameters		
	5	3	2
Control	75%	75%	57%
Isoproterenol	63%	73%	54%
Propranolol	77%	80%	27%

## Discussion

We selected the TreeBagger algorithm for classification because it provides a relatively high accuracy of classification and ease of interpretation when compared to other supervised machine learning approaches [3]. The input parameters and classification levels for these data are relatively simple from a machine learning standpoint. Thus, the larger memory requirement employed by TreeBagger is not significant given the improved accuracy of classification and interpretability of the results. Future iterations involving longitudinal parameters and expanded sets of training data may make the memory costs more significant. However, the accuracy of classification gleaned from TreeBagger may still be the most important factor when compared to the poor accuracy of existing pre-clinical cardiac drug screening techniques [27]. This algorithm is especially robust when we consider that we are able to predict the drug treatment using only data from a single beat of one hiPS-CM membrane with over 70% accuracy. When this predictive power is applied over the length of an entire recording of a membrane (1 min long containing 50–100 beat waveforms), the treatment was correctly identified 100% of the time. For the practical purposes of using hiPS-CM as a drug screening model, data for multiple beats and many cell membranes will be collected, even in a high throughput model. With these repeated temporal and population wide measures, our confidence in the predictive power of the learned algorithm increases significantly. In general, the use of supervised learning allows for data-driven prediction and classification of unknown drug treatments [28]. Unknown drugs can be classified by simple comparison of their effect on specific parameters, but only one-dimensionally on a parameter by parameter basis. Supervised learning augments these individual parameter comparisons by synthesizing all parameter data from multiple drug treatments and determining the relative contribution of each parameter to the classification of each drug. This is important as many drugs have effects on multiple parameters and to varying degrees. Further, supervised learning incorporates statistical modelling that provides probabilities and confidence of classification based on the aggregation of all data from all parameters measured and all treatments performed.

Our model reduction determined by the TreeBagger algorithm (upslope and downslope were the least relevant parameters and were removed in the simplified 3 parameter model) is consistent with our a priori knowledge of the mechanism of propranolol and isoproterenol. As mentioned previously, modulation of calcium transport with propranolol and isoproterenol would be expected to affect the plateau height and width of the depolarization waveform as was determined by our algorithm. The upslope of a cardiac action potential is primarily driven by the fast sodium channels and the down slope is driven by a combination of delayed rectifier potassium channels (I_KS_, I_KR_, I_K1_). Propranolol and isoproterenol would not be expected to affect the upslope or downslope of the cardiac action potential waveform since they do not affect the fast sodium or delayed rectifier channels. Although we did observe significant changes in both upslope and downslope due to these β–adrenergic agents, our algorithm accurately placed the lowest significance on upslope and downslope as metrics for determining drug treatment classification.

The most common misclassification was a false positive classification of control cells as isoproterenol. Despite using a concentration of isoproterenol two orders of magnitude larger than the EC_50_ for hiPS-CMs derived from the wtc11 hiPS line, the effect on the beat rate and the peak width was still significantly smaller than cells treated with propranolol at only one order of magnitude larger than the IC_50_. Although all other metrics were affected to a greater degree by isoproterenol than propranolol, the misclassification of individual peaks as isoproterenol is likely due to the diminished effects on peak width.

All of these data were obtained optically with near-infrared light and thus in a non-invasive and non-destructive manner, using 2-photon microscopy. 2-photon microscopy was utilized over standard epi-fluorescence because the z-plane resolution obtained using 2-photon provides sharper spatial resolution of membrane fluorescence providing improved SNR and membrane motion tracking. Although not exploited here, there is potential for assessing the chronic effects of drugs on hiPS-CM in a longitudinal fashion in vitro. The signal to noise ratio (SNR) of data captured using 2-photon microscopy from hemicyanine VSDs, such as di-4-ANE(F)PPTEA, and its sensitivity to changes in voltage (typically on the order of 10–20% ΔF/F per 100mV [13]) is more than sufficient for capturing the depolarization waveform features necessary for training the learning algorithm. The emission kinetics of the hemicyanine VSDs are very sensitive to rapid voltage changes and are limited only by the underlying physiological processes since the electrochromic effect of these dyes is produced by a direct interaction of the electric field with the chromophore [13].

Potential limitations of using VSDs to assess the effects of cardiotoxic drugs exist. The speed of acquisition required to capture the spatiotemporal resolution of the VSD signal necessary to detect subtle changes in the action potential waveform is high. Line scans provide the speed required to acquire high resolution data from multiple adjacent cellular membranes. This allows quality data from small groups of cells. Larger scale tissue and organ level effects could be pieced together using a series of line scans; however, this requires larger acquisition times which will compromise the quality of rapid events such as membrane depolarization. Additionally, the VSD data from isoproterenol-treated cells exhibited a substantial increase in upslope speed and max height (Fig 3B), as well as occasional arrhythmias. While arrhythmias have been reported in previous studies using isoproterenol [29], the change in upslope speed and max height contradicts data collected using manual patch clamp in which no change in either parameter is observed. hiPS-derived cardiomyocytes exhibit a neonatal phenotype until approximately 50 days after differentiation with respect to their ion channel expression (reduced rectifying K1 channels and fast Na channels) [30]. Despite varying channel expression, isoproterenol is still not expected to have any effect on the fast sodium channels that drive the upslope and max height of depolarization, even in naïve Day 33 hiPS-derived cardiomyocytes, such as those used in this study. This discrepancy highlights the need to further test VSDs in future studies as a surrogate for electrophysiological measurements in a direct comparison with gold standard methods, such as patch clamp. However, the data presented demonstrate that these cells, despite their immaturity and presence of arrhythmias in certain conditions, are capable of responding to the β–adrenergic drugs administered in this study in a detectable and repeatable fashion using VSDs, even if that response contradicts other electrophysiological methods.

Future work will focus on improving the predictive and discovery power of the trained algorithm to identify mechanisms of electrophysiological modulation. Two potential pathways exist for extending the predictive power of a machine learning approach. First, a library of depolarization waveforms from cells exposed to drugs with known mechanisms of cardiac effects can be collected and incorporated into the training set. As the knowledge base of training data grows, the more refined and sophisticated the classification of unknown data becomes. When assessing novel drugs, this approach would allow comparison to the existing library of drugs. For example, a novel drug may exhibit propranolol-like effects, providing direction as to how to pursue the study of potential mechanisms of the cardiac effects caused by the novel drug (given that we know the mechanism that leads to the functional alteration of the electrophysiology of each drug in the database).

A second pathway for extending the predictive power of machine learning for electrophysiology is to pursue more unsupervised learning approaches. In this regime, rather than defining the parameters that we think are important in the waveform due to our a priori understanding of channel mechanics in electrophysiology, we let the algorithm define the waveform features that are most significant in delineating between treatment groups. We can also improve upon the relatively arbitrary metrics utilized here (i.e. upslope, plateau height, etc.) and instead move towards a more mechanistic classification of waveform modulation (i.e. I_Na_ channel modulation, I_kr_ channel modulation, etc.) in both supervised and unsupervised learning approaches.

The overall goal of this approach and application of machine learning is to accelerate drug development through more rapid and efficient pre-clinical testing modalities. Using VSD and other novel methods of electrophysiological assessment of hiPS-CM, we can rapidly generate meaningful data regarding the human specific electrophysiological effects of drugs on a sub-cellular scale. The predictive power of machine learning can then be used to maximize these data’s utility as a drug discovery platform and be used to identify potential side effects that previously have gone undetected in other pre-clinical testing platforms.

## Supporting Information

S1 Fig Isoproterenol and Propranolol Beat Rate Dose Response Curves. The EC_50_ and IC_50_ of isoproterenol and propranolol, were determined to be 10^−9.1^ M and 10^−5.9^ M, respectively, in wtc11 hiPS-CMs. Data are mean +/- SD of N = 6 (isoproterenol) and N = 3 (propranolol) spontaneously beating clusters of hiPS-CMs. Fitted curves are logistic regression with R^2^ = 0.947 and R^2^ = 0.954 for isoproterenol and propranolol, respectively.
(TIF)

S2 Fig Motion Artifact Correction. Motion artifact resulting from the spontaneous beating of cell clusters was compensated for in data post-processing using a Gaussian tracking and correction algorithm. Quantification of membrane 3 depolarization peaks using pre-corrected raw data correlates well with membrane 1 depolarization peaks quantified using data corrected with the Gaussian tracking algorithm.
(TIF)

S3 Fig Photobleaching Correction. Photobleaching was accounted for by fitting a second order exponential (y = ae^bx^ + ce^dx^) and subtracting from the signal. Top panel: raw signal collected by the instrument. Middle panel: overlay of second order exponential fit and raw signal. Bottom panel: Resultant signal after subtracting the exponential fit and normalizing to baseline fluorescence of the membrane (F_o_).
(TIF)

S1 Code MATLAB Scripts. 3 custom scripts were used for data analysis. The first script was for photobleaching correction. The second script performed waveform detection and parameter quantification. The final script trained the TreeBagger algorithm and validated algorithm performance.
(DOCX)
