# Supplementary figures and images for: Supervised Machine Learning for Classification of the Electrophysiological Effects of Chronotropic Drugs on Human Induced Pluripotent Stem Cell-Derived Cardiomyocytes
Source: PLoS One. 2015 Dec 22;10(12):e0144572. doi: 10.1371/journal.pone.0144572 (PMC4690607; doi:10.1371/journal.pone.0144572)

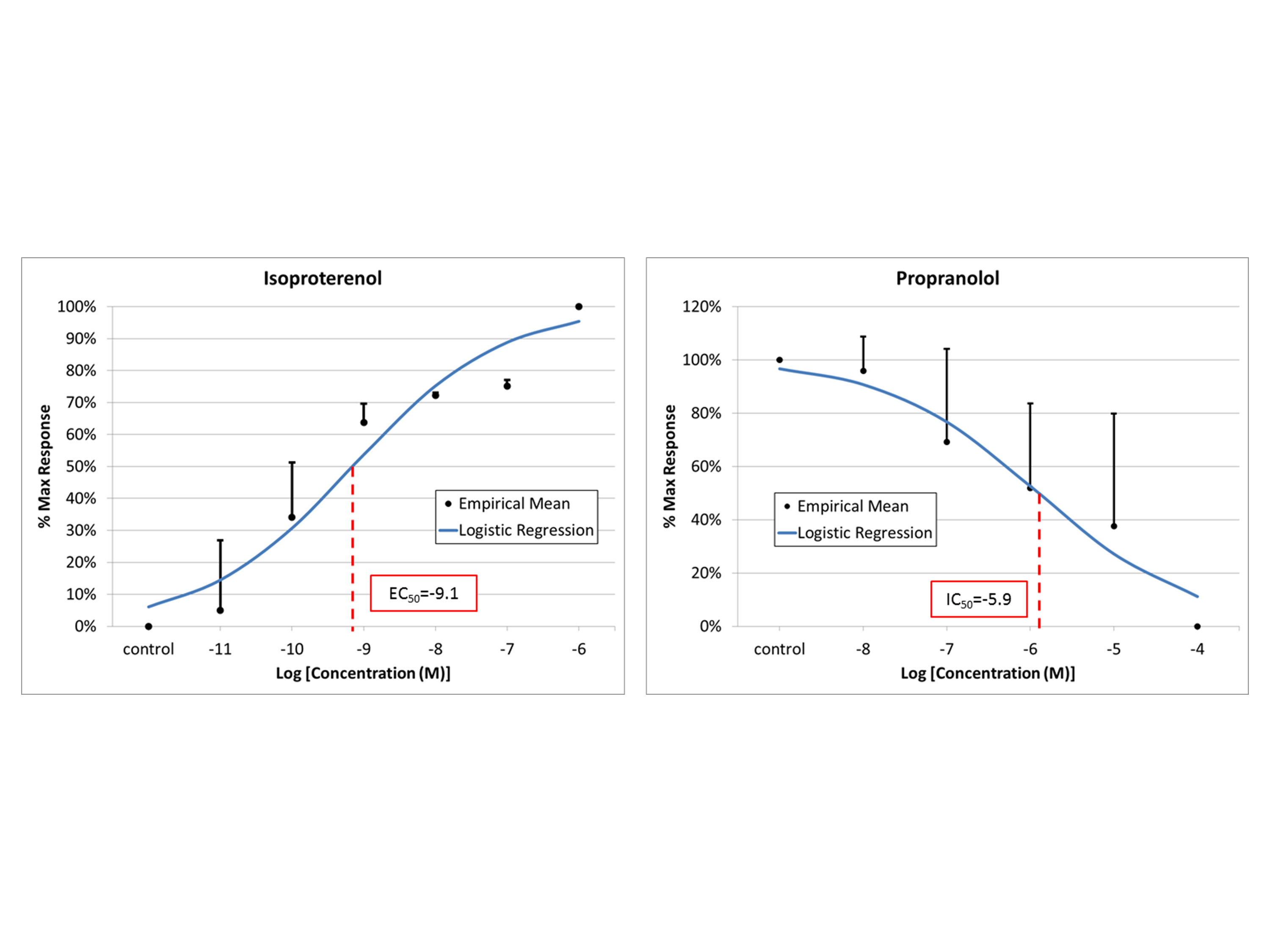

Supplement: S1 Fig — The EC50 and IC50 of isoproterenol and propranolol, were determined to be 10−9.1 M and 10−5.9 M, respectively, in wtc11 hiPS-CMs. Data are mean +/- SD of N = 6 (isoproterenol) and N = 3 (propranolol) spontaneously beating clusters of hiPS-CMs. Fitted curves are logistic regression with R2 = 0.947 and R2 = 0.954 for isoproterenol and propranolol, respectively. (TIF) [file pone.0144572.s001.tif]

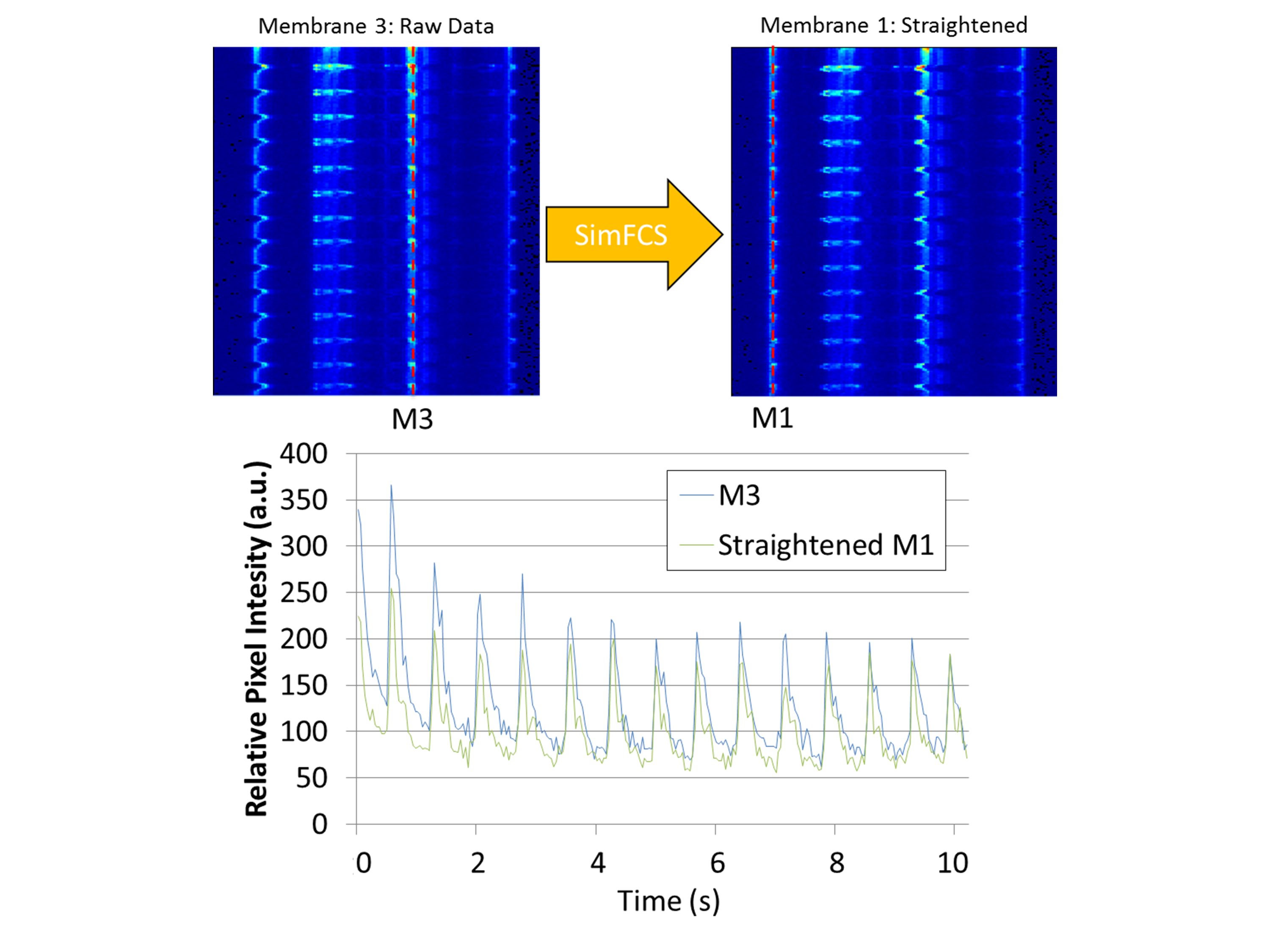

Supplement: S2 Fig — Motion artifact resulting from the spontaneous beating of cell clusters was compensated for in data post-processing using a Gaussian tracking and correction algorithm. Quantification of membrane 3 depolarization peaks using pre-corrected raw data correlates well with membrane 1 depolarization peaks quantified using data corrected with the Gaussian tracking algorithm. (TIF) [file pone.0144572.s002.tif]

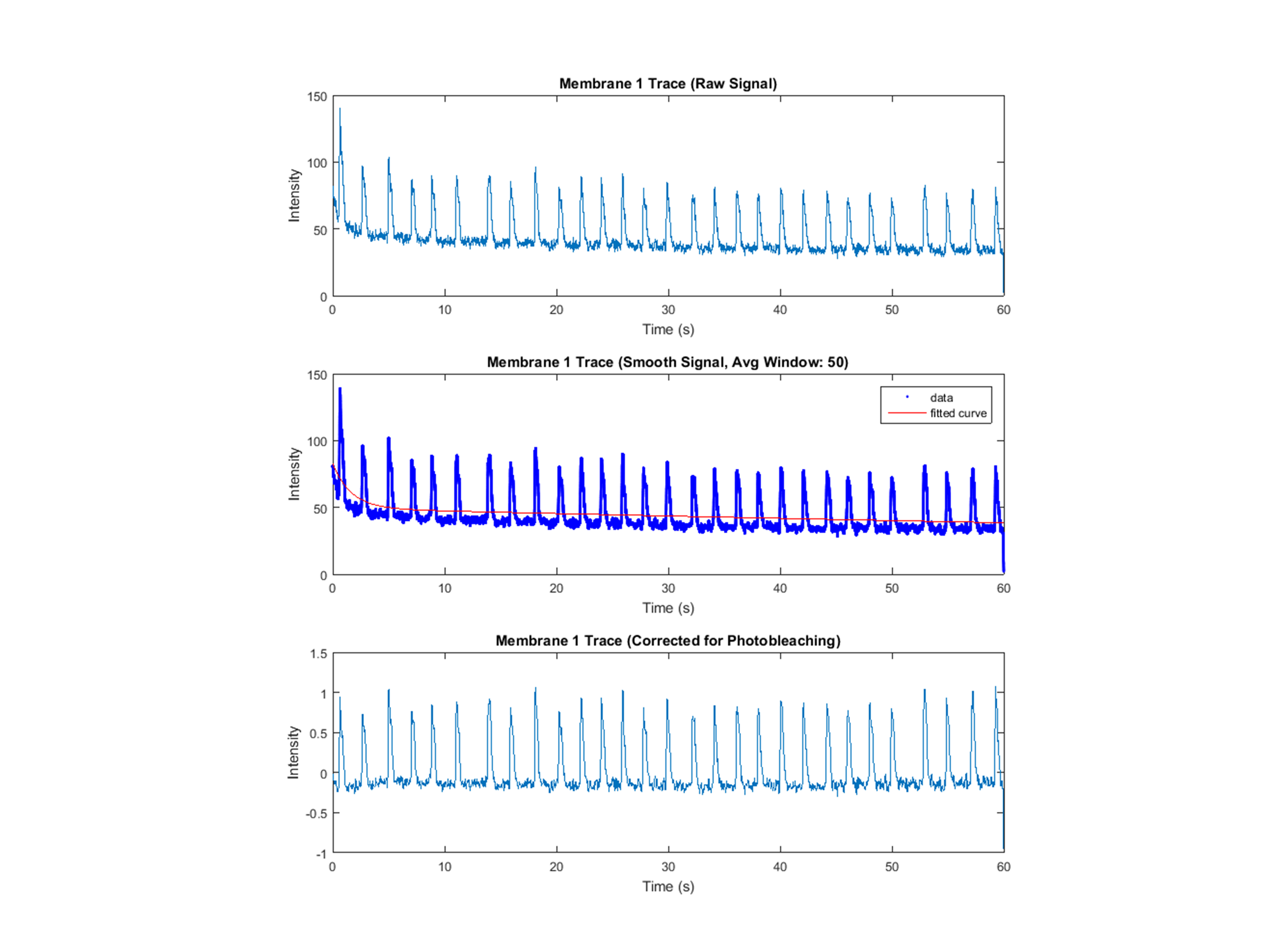

Supplement: S3 Fig — Photobleaching was accounted for by fitting a second order exponential (y = aebx + cedx) and subtracting from the signal. Top panel: raw signal collected by the instrument. Middle panel: overlay of second order exponential fit and raw signal. Bottom panel: Resultant signal after subtracting the exponential fit and normalizing to baseline fluorescence of the membrane (Fo). (TIF) [file pone.0144572.s003.tif]
