# Supplementary material for: Supervised Machine Learning for Classification of the Electrophysiological Effects of Chronotropic Drugs on Human Induced Pluripotent Stem Cell-Derived Cardiomyocytes
Source: PLoS One. 2015 Dec 22;10(12):e0144572. doi: 10.1371/journal.pone.0144572 (PMC4690607; doi:10.1371/journal.pone.0144572)
Supplement: S1 Code — 3 custom scripts were used for data analysis. The first script was for photobleaching correction. The second script performed waveform detection and parameter quantification. The final script trained the TreeBagger algorithm and validated algorithm performance. (DOCX) [file pone.0144572.s004.docx]

**Photobleaching Correction**

% Photobleaching correction and normalization of raw data

close all

clear all

clc

filename = uigetfile;

uiimport(filename);

pause

% Averaging Window

z=50;

B=Series5;

C=smooth(B,z);

for i=1:length(Series6)

D(i)=i*6e-04;

end

D=D';

figure

subplot(3,4,[1 2])

plot(D,B)

title('Membrane 1 Trace (Raw Signal)')

xlabel('Time (s)')

ylabel('Intensity')

subplot(3,4,[5 6])

f = fit(D,C,'exp2');

f_y = feval(f,D);

corrected=(C-f_y)./f_y;

plot(f,D,C)

title(['Membrane 1 Trace (Smooth Signal, Avg Window: ' num2str(z) ')'])

xlabel('Time (s)')

ylabel('Intensity')

subplot(3,4,[9 10])

plot(D,corrected)

title('Membrane 1 Trace (Corrected for Photobleaching)')

xlabel('Time (s)')

ylabel('Intensity')

B2=Series6;

C2=smooth(B2,50);

subplot(3,4,[3 4])

plot(D,B2)

title('Membrane 2 Trace (Raw Signal)')

xlabel('Time (s)')

ylabel('Intensity')

subplot(3,4,[7 8])

f = fit(D,C2,'exp2');

f_y = feval(f,D);

corrected2=(C2-f_y)./f_y;

plot(f,D,C2)

title(['Membrane 2 Trace (Smooth Signal, Avg Window: ' num2str(z) ')'])

xlabel('Time (s)')

ylabel('Intensity')

subplot(3,4,[11 12])

plot(D,corrected2)

title('Membrane 2 Trace (Corrected for Photobleaching)')

xlabel('Time (s)')

ylabel('Intensity')

for q=1:4

filename(end)=[];

end

uisave({'corrected','corrected2','D'},[filename '_corrected']);

**Waveform Detection and Paramter Quantification**

close all

clear all

clc

% open file chooser to select data file (must be .mat file with your data stored as a variable named 'm3')

filename = uigetfile;

open(filename);

prompt = 'Membrane (1 or 2)? ';

mem = input(prompt);

if mem==1

noisyECG_withTrend= ans.corrected;

else

noisyECG_withTrend= ans.corrected2;

end

ECG_data = noisyECG_withTrend;

for q=1:4

filename(end)=[];

end

filename=[filename '_membrane_' num2str(mem)];

% real time scale

time = ans.D;

% arbitrary time scale

t = 1:length(noisyECG_withTrend);

% plot raw data

correctedplot = figure;

subplot(3,2,1)

plot(time,noisyECG_withTrend)

title('Detrended VSD Signal')

xlabel('Time (s)'); ylabel('Fluorescence Intensity (a.u.)')

grid on

ParaPeakHeight= 5;

ParaTroughHeight= 2;

ParaPeakDistance= .5;

ParaTroughDistance= .5;

ParaPeakDistance=round(ParaPeakDistance*max(t)/max(time));

ParaTroughDistance=round(ParaTroughDistance*max(t)/max(time));

% find peaks

[~,locs_Rwave] = findpeaks(ECG_data,'MinPeakHeight',ParaPeakHeight,'MinPeakDistance',ParaPeakDistance);

% flip data and find troughs

ECG_inverted = -ECG_data;

[~,locs_Swave] = findpeaks(ECG_inverted,'MinPeakHeight',ParaTroughHeight, 'MinPeakDistance',ParaTroughDistance);

% convert min and max locations to real time scale for plotting

locs_Rwave_convert=locs_Rwave*ans.D(1);

locs_Swave_convert=locs_Swave*ans.D(1);

% plot identified peaks and troughs

subplot(3,2,2)

plot(time,ECG_data);

hold on

plot(locs_Rwave_convert,ECG_data(locs_Rwave),'rv','MarkerFaceColor','r');

hold on

plot(locs_Swave_convert,ECG_data(locs_Swave),'rs','MarkerFaceColor','b');

grid on;

legend('Detrended VSD Signal','Peak','Trough');

xlabel('Time (s)'); ylabel('Fluorescence Intensity (a.u.)')

title('Peak and Trough Identification')

% 1) select longer of 2 arrays: peak or trough, run loop for length of that

% array

% 2) test if signal starts with peak or array

% 3) progress through array and test for alternating single peaks and

% arrays (2 seperate tests per loop--one for troughs, one for peaks)

% 4) if multiple peak/trough encountered, find local max/min and delete

% other redundant peaks/troughs

% 5) resume checking alternating peak troughs with updated arrays until next error encountered

% Notes:

% Length of indexing array running loop will dynamically change (the length

% will be adjusted as redundant peaks/troughs are removed)

corrections=0; % initialize counters of corrections

deletedpeaks=0;

deletedtroughs=0;

count=0;

lengthRwaveinit= length(locs_Rwave);

lengthSwaveinit= length(locs_Swave);

lengthTwaveinit= length(locs_Rwave)+length(locs_Swave);

if length(locs_Rwave) > length(locs_Swave) % lenght of peak locations array is longer

for i=1:length(locs_Swave)-1 % itierate loops for length of shorter array (trough array)

if locs_Rwave(i)==locs_Rwave(end-1) %loop termination to account for trough or peak ending

break

end

if locs_Swave(i)==locs_Swave(end-1) %loop termination to account for trough or peak ending

break

end

if locs_Rwave(1) > locs_Swave(1)

locs_Swave(1)=[];

end

while locs_Rwave(i+1) < locs_Swave(i) % check for multiple peaks (iterate until next trough is reached)

count=count+1; % for debugging reference

if ECG_data(locs_Rwave(i)) < ECG_data(locs_Rwave(i+1)) % compare intensity values of repeated peaks

locs_Rwave(i)=[]; % delete smaller peak

corrections= corrections+1;

deletedpeaks= deletedpeaks+1;

else

locs_Rwave(i+1)=[]; % delete smaller peak

corrections= corrections+1;

deletedpeaks= deletedpeaks+1;

end

end

while locs_Swave(i+1) < locs_Rwave(i+1) % check for multiple troughs (iterate until next peak is reached)

if -ECG_data(locs_Swave(i)) < -ECG_data(locs_Rwave(i+1)) % compare intensity values of repeated inverted troughs

locs_Swave(i)=[]; % delete smaller trough

corrections= corrections+1;

deletedtroughs= deletedtroughs+1;

else

locs_Swave(i+1)=[]; % delete smaller trough

corrections= corrections+1;

deletedtroughs= deletedtroughs+1;

end

end

end

else

for i=1:length(locs_Rwave)-1 % itierate loops for length of shorter array (peak array)

count=count+1; % for debugging reference

if locs_Rwave(i)==locs_Rwave(end-1) %loop termination to account for trough or peak ending

break

end

if locs_Swave(i)==locs_Swave(end-1) %loop termination to account for trough or peak ending

break

end

if locs_Rwave(1) > locs_Swave(1)

locs_Swave(1)=[];

end

while locs_Rwave(i+1) < locs_Swave(i) % check for multiple peaks (iterate until next trough is reached)

count=count+1; % for debugging reference

if ECG_data(locs_Rwave(i)) < ECG_data(locs_Rwave(i+1)) % compare intensity values of repeated peaks

locs_Rwave(i)=[]; % delete smaller peak

corrections= corrections+1;

deletedpeaks= deletedpeaks+1;

else

locs_Rwave(i+1)=[]; % delete smaller peak

corrections= corrections+1;

deletedpeaks= deletedpeaks+1;

end

end

while locs_Swave(i+1) < locs_Rwave(i+1) % check for multiple troughs (iterate until next peak is reached)

count=count+1; % for debugging reference

if -ECG_data(locs_Swave(i)) < -ECG_data(locs_Rwave(i+1)) % compare intensity values of repeated inverted troughs

locs_Swave(i)=[]; % delete smaller trough

corrections= corrections+1;

deletedtroughs= deletedtroughs+1;

else

locs_Swave(i+1)=[]; % delete smaller trough

corrections=corrections+1;

deletedtroughs= deletedtroughs+1;

if locs_Rwave(i)== locs_Rwave(end) %loop termination to account for trough or peak ending

break

end

if locs_Swave(i)== locs_Swave(end) %loop termination to account for trough or peak ending

break

end

end

end

end

end

i=1; % reset i

fprintf('Number of Corrections: %d \n', corrections);

percorr= corrections/lengthTwaveinit*100;

fprintf('Percentage of Corrections: %d \n \n', percorr);

fprintf('Number of Peak Corrections: %d \n', deletedpeaks);

peakpercorr= deletedpeaks/lengthRwaveinit*100;

fprintf('Percentage of Peak Corrections: %d \n \n', peakpercorr)

fprintf('Number of Trough Corrections: %d \n', deletedtroughs);

troughpercorr= deletedtroughs/lengthSwaveinit*100;

fprintf('Percentage of Trough Corrections: %d \n \n', troughpercorr);

% reconvert corrected min and max locations to real time scale for plotting

locs_Rwave_convert=locs_Rwave*ans.D(1);

locs_Swave_convert=locs_Swave*ans.D(1);

% plot corrected peaks and troughs

subplot(3,2,3)

hold on

plot(time,ECG_data);

plot(locs_Rwave_convert,ECG_data(locs_Rwave),'rv','MarkerFaceColor','r');

plot(locs_Swave_convert,ECG_data(locs_Swave),'rs','MarkerFaceColor','b');

grid on;

legend('Detrended VSD Signal','Peak','Trough');

xlabel('Time (s)'); ylabel('Fluorescence Intensity (a.u.)')

title('Corrected Peak and Trough Identification')

% create arrays of peak and trough values

maximum=[ECG_data(locs_Rwave)];

minimum=[ECG_data(locs_Swave)];

% calculate beat rate (BR)

BR=length(maximum)/max(time);

fprintf('The Beat Rate is: %d beats/s \n', BR)

% find slope of curve at all points

slope=diff(ECG_data)./diff(time);

% calculate metrics at each depolarization event

% if signal starts with a peak

if locs_Swave(1)>locs_Rwave(1)

for i=1:(length(minimum)-2)

% find indices of all values that fall between trough (i) and peak (i+1)

j=find(minimum(i)<ECG_data & ECG_data<maximum(i+1));

% find index of point closest to the peak on upslope

k=find(locs_Swave(i)<j & j<locs_Rwave(i+1),1,'last');

% find indices of all values that fall between peak (i+1) and trough (i+1)

l=find(minimum(i+1)<ECG_data & ECG_data<maximum(i+1));

% find index of point closest to the peak on downslope

m=find(locs_Rwave(i+1)<l & l<locs_Swave(i+1),1,'first');

% location of upslope

upslope_loc(i)=j(k);

% value at upslope location

upslope_value(i)=ECG_data(j(k));

% location downslope

downslope_loc(i)=l(m);

% value at downslope location

downslope_value(i)=ECG_data(l(m));

% upslope value

mup(i)=slope(j(k));

% downslope value

mdown(i)=slope(l(m));

% calculate half of maximum value

n=(maximum(i+1)-minimum(i))/2+minimum(i);

% find indices of all values that fall between trough (i) and peak (i+1)

o=find(locs_Swave(i)<=t & t<=locs_Rwave(i+1));

% find values between trough (i) and peak (i+1)

localupECG_data=ECG_data(locs_Swave(i):locs_Rwave(i+1));

% find index of value between trough (i) and peak (i+1) closest to half of max value

[idx,D]=knnsearch(localupECG_data,n);

% location of half of max value on upslope

up50_loc(i)=(t(o(idx)));

% half of max value on upslope

up50_value(i)=localupECG_data(idx);

% find indices of all values that fall between peak (i+1) and trough (i+1)

p=find(locs_Rwave(i+1)<=t & t<=locs_Swave(i+1));

% find values between peak (i+1) and trough (i+1)

localdownECG_data=ECG_data(locs_Rwave(i+1):locs_Swave(i+1));

% find index of value between peak (i+1) and trough (i+1) closest to half of max value

[idx,D]=knnsearch(localdownECG_data,n);

% location of half of max value on downslope

down50_loc(i)=(t(p(idx)));

% half of max value on downslope

down50_value(i)=localdownECG_data(idx);

% calculate width @ half max height

w(i)= down50_loc(i)-up50_loc(i);

% plateau location at midpoint of half max height locations

hplateau_loc(i)=round((up50_loc(i)+w(i)/2));

% pleateu height value

hplateau_value(i)=ECG_data(round((up50_loc(i)+w(i)/2)));

end

% if signal starts with a trough

elseif locs_Swave(1)<locs_Rwave(1)

for i=1:(length(maximum)-2)

% find indices of all values that fall between trough (i) and peak (i)

j=find(minimum(i)<ECG_data & ECG_data<maximum(i));

% find index of point closest to the peak on upslope

k=find(locs_Swave(i)<j & j<locs_Rwave(i),1,'last');

% find indices of all values that fall between peak (i) and trough (i+1)

l=find(minimum(i+1)<ECG_data & ECG_data<maximum(i));

% find index of point closest to the peak on downslope

m=find(locs_Rwave(i)<l & l<locs_Swave(i+1),1,'first');

% location of upslope

upslope_loc(i)=j(k);

% value at upslope location

upslope_value(i)=ECG_data(j(k));

% location downslope

downslope_loc(i)=l(m);

% value at downslope location

downslope_value(i)=ECG_data(l(m));

% upslope value

mup(i)=slope(j(k));

% downslope value

mdown(i)=slope(l(m));

% calculate half of maximum value

n=(maximum(i)-minimum(i))/2+minimum(i);

% find indices of all values that fall between trough (i) and peak (i)

o=find(locs_Swave(i)<=t & t<=locs_Rwave(i));

% find values between trough (i) and peak (i)

localupECG_data=ECG_data(locs_Swave(i):locs_Rwave(i));

% find index of value between trough (i) and peak (i) closest to half of max value

[idx,D]=knnsearch(localupECG_data,n);

% location of half of max value on upslope

up50_loc(i)=(t(o(idx)));

% half of max value on upslope

up50_value(i)=localupECG_data(idx);

% find indices of all values that fall between peak (i) and trough (i+1)

p=find(locs_Rwave(i)<=t & t<=locs_Swave(i+1));

% find values between peak (i) and trough (i+1)

localdownECG_data=ECG_data(locs_Rwave(i):locs_Swave(i+1));

% find index of value between peak (i) and trough (i+1) closest to half of max value

[idx,D]=knnsearch(localdownECG_data,n);

% location of half of max value on downslope

down50_loc(i)=(t(p(idx)));

% half of max value on downslope

down50_value(i)=localdownECG_data(idx);

% calculate width @ half max height

w(i)= down50_loc(i)- up50_loc(i);

% plateau location at midpoint of half max height locations

hplateau_loc(i)=round((up50_loc(i)+w(i)/2));

% pleateu height value

hplateau_value(i)=ECG_data(round((up50_loc(i)+w(i)/2)));

end

% if number of troughs and peaks are equal

else

% if signal starts with a trough

if locs_Swave(i)<locs_Rwave(i)

for i=1:(length(minimum)-2)

% find indices of all values that fall between trough (i) and peak (i)

j=find(minimum(i)<ECG_data & ECG_data<maximum(i));

% find index of point closest to the peak on upslope

k=find(locs_Swave(i)<j & j<locs_Rwave(i),1,'last');

% find indices of all values that fall between peak (i) and trough (i+1)

l=find(minimum(i+1)<ECG_data & ECG_data<maximum(i));

% find index of point closest to the peak on downslope

m=find(locs_Rwave(i)<l & l<locs_Swave(i+1),1,'first');

% location of upslope

upslope_loc(i)=j(k);

% value at upslope location

upslope_value(i)=ECG_data(j(k));

% location downslope

downslope_loc(i)=l(m);

% value at downslope location

downslope_value(i)=ECG_data(l(m));

% upslope value

mup(i)=slope(j(k));

% downslope value

mdown(i)=slope(l(m));

% calculate half of maximum value

n=(maximum(i)-minimum(i))/2+minimum(i);

% find indices of all values that fall between trough (i) and peak (i)

o=find(locs_Swave(i)<=t & t<=locs_Rwave(i));

% find values between trough (i) and peak (i)

localupECG_data=ECG_data(locs_Swave(i):locs_Rwave(i));

% find index of value between trough (i) and peak (i) closest to half of max value

[idx,D]=knnsearch(localupECG_data,n);

% location of half of max value on upslope

up50_loc(i)=(t(o(idx)));

% half of max value on upslope

up50_value(i)=localupECG_data(idx);

% find indices of all values that fall between peak (i) and trough (i+1)

p=find(locs_Rwave(i)<=t & t<=locs_Swave(i+1));

% find values between peak (i) and trough (i+1)

localdownECG_data=ECG_data(locs_Rwave(i):locs_Swave(i+1));

% find index of value between peak (i) and trough (i+1) closest to half of max value

[idx,D]=knnsearch(localdownECG_data,n);

% location of half of max value on downslope

down50_loc(i)=(t(p(idx)));

% half of max value on downslope

down50_value(i)=localdownECG_data(idx);

% calculate width @ half max height

w(i)= down50_loc(i)-up50_loc(i);

% plateau location at midpoint of half max height locations

hplateau_loc(i)=round((up50_loc(i)+w(i)/2));

% pleateu height value

hplateau_value(i)=ECG_data(round((up50_loc(i)+w(i)/2)));

end

% if signal starts with a peak

else

for i=1:(length(maximum)-2)

% find indices of all values that fall between trough (i) and peak (i+1)

j=find(minimum(i)<ECG_data & ECG_data<maximum(i+1));

% find index of point closest to the peak on upslope

k=find(locs_Swave(i)<j & j<locs_Rwave(i+1),1,'last');

% find indices of all values that fall between peak (i+1) and trough (i+1)

l=find(minimum(i+1)<ECG_data & ECG_data<maximum(i+1));

% find index of point closest to the peak on downslope

m=find(locs_Rwave(i+1)<l & l<locs_Swave(i+1),1,'first');

% location of upslope

upslope_loc(i)=j(k);

% value at upslope location

upslope_value(i)=ECG_data(j(k));

% location downslope

downslope_loc(i)=l(m);

% value at downslope location

downslope_value(i)=ECG_data(l(m));

% upslope value

mup(i)=slope(j(k));

% downslope value

mdown(i)=slope(l(m));

% calculate half of maximum value

n=(maximum(i+1)-minimum(i))/2+minimum(i);

% find indices of all values that fall between trough (i) and peak (i+1)

o=find(locs_Swave(i)<=t & t<=locs_Rwave(i+1));

% find values between trough (i) and peak (i+1)

localupECG_data=ECG_data(locs_Swave(i):locs_Rwave(i+1));

% find index of value between trough (i) and peak (i+1) closest to half of max value

[idx,D]=knnsearch(localupECG_data,n);

% location of half of max value on upslope

up50_loc(i)=(t(o(idx)));

% half of max value on upslope

up50_value(i)=localupECG_data(idx);

% find indices of all values that fall between peak (i+1) and trough (i+1)

p=find(locs_Rwave(i+1)<=t & t<=locs_Swave(i+1));

% find values between peak (i+1) and trough (i+1)

localdownECG_data=ECG_data(locs_Rwave(i+1):locs_Swave(i+1));

% find index of value between peak (i+1) and trough (i+1) closest to half of max value

[idx,D]=knnsearch(localdownECG_data,n);

% location of half of max value on downslope

down50_loc(i)=(t(p(idx)));

% half of max value on downslope

down50_value(i)=localdownECG_data(idx);

% calculate width @ half max height

w(i)= down50_loc(i)-up50_loc(i);

% plateau location at midpoint of half max height locations

hplateau_loc(i)=round((up50_loc(i)+w(i)/2));

% pleateu height value

hplateau_value(i)=ECG_data(round((up50_loc(i)+w(i)/2)));

end

end

end

% calculate stats for each metric

mean_hmax=mean(maximum)-mean(ECG_data);

std_hmax=std(maximum);

se_hmax=std(maximum)/sqrt(length(maximum));

mean_upslope=mean(mup);

std_upslope=std(mup);

se_upslope=std(mup)/sqrt(length(mup));

mean_downslope=mean(mdown);

std_downslope=std(mdown);

se_downslope=std(mdown)/sqrt(length(mdown));

mean_w=mean(w);

std_w=std(w);

se_w=std(w)/sqrt(length(w));

mean_hplateau=mean(hplateau_value)-mean(ECG_data);

std_hplateau=std(hplateau_value);

se_hplateau=std(hplateau_value)/sqrt(length(hplateau_value));

% plot slope identification points

subplot(3,2,4)

hold on

plot(t,ECG_data);

plot(upslope_loc,upslope_value,'rv','MarkerFaceColor','r');

plot(downslope_loc,downslope_value,'rs','MarkerFaceColor','b');

grid on;

legend('Detrended VSD Signal','Upslope','Downslope');

xlabel('Time (s)'); ylabel('Fluorescence Intensity (a.u.)')

title('Slope Identification')

% plot width identification points

subplot(3,2,5)

hold on

plot(t,ECG_data);

plot(up50_loc,up50_value,'rv','MarkerFaceColor','r');

plot(down50_loc,down50_value,'rs','MarkerFaceColor','b');

grid on;

legend('Detrended VSD Signal','Up50','Down50');

xlabel('Time (s)'); ylabel('Fluorescence Intensity (a.u.)')

title('Width Identification')

% plot plateau identification points

subplot(3,2,6)

hold on

plot(t,ECG_data);

plot(hplateau_loc,hplateau_value,'rv','MarkerFaceColor','r');

grid on;

legend('Detrended VSD Signal','hplateau');

xlabel('Time (s)'); ylabel('Fluorescence Intensity (a.u.)')

title('Plateau Height Identification')

saveas(correctedplot,[filename '_plot'], 'fig');

while length(maximum)>length(mup)

maximum(end)=[];

end

% create dataset for TreeBagger input

% request treatment tag

filename

prompt = 'Name of Treatment? \n 1=propranolol \n 2=isoproterenol \n 3=control \n';

gate = input(prompt);

if gate==1

name='propranolol';

elseif gate==2

name='isoproterenol';

else

name='control';

end

name

for i = 1:(length(maximum))

treatment{i}=name;

end

mup=mup'; mdown=mdown'; w=w'; hplateau_value=hplateau_value'; treatment=treatment';

ds = dataset(maximum,mup,mdown,w,hplateau_value,treatment);

ds.Properties.VarNames(:);

uisave('ds',[filename '_dataset']);

%Report Generation

filenamereport= [filename '_report'];

sheet= 1;

xltag1= {'Mean'};

rangetag1= 'A2';

xlswrite(filenamereport,xltag1,sheet,rangetag1);

xltag2= {'Std Dev'};

rangetag2= 'A3';

xlswrite(filenamereport,xltag2,sheet,rangetag2);

xltag3= {'Std Error'};

rangetag3= 'A4';

xlswrite(filenamereport,xltag3,sheet,rangetag3);

widthplat= {'Upslope','Downslope','Width','Plateau';};

rangevar= 'B1';

xlswrite(filenamereport,widthplat,sheet,rangevar);

aqpara= {'Peak Height','Peak Distance', 'Trough Height', 'Trough Distance'; ParaPeakHeight,ParaPeakDistance,ParaTroughHeight,ParaTroughDistance};

rangeaqpara= 'A7';

xlswrite(filenamereport,aqpara,sheet,rangeaqpara);

xldata= {mean_upslope,mean_downslope,mean_w,mean_hplateau};

rangesdat= 'B2';

xlswrite(filenamereport,xldata,sheet,rangesdat);

xldata2= {std_upslope,std_downslope,std_w,std_hplateau};

rangesdat2= 'B3';

xlswrite(filenamereport,xldata2,sheet,rangesdat2);

xldata3= {se_upslope,se_downslope,se_w,se_hplateau};

rangesdat3= 'B4';

xlswrite(filenamereport,xldata3,sheet,rangesdat3);

xldata4= {'Peak','Trough','Total'};

rangesdat4= 'B10';

xlswrite(filenamereport,xldata4,sheet,rangesdat4);

xldata5= {'Totals',deletedpeaks,deletedtroughs,corrections};

rangesdat5= 'A11';

xlswrite(filenamereport,xldata5,sheet,rangesdat5);

xldata6= {'Percents',peakpercorr,troughpercorr,percorr};

rangesdat6= 'A12';

xlswrite(filenamereport,xldata6,sheet,rangesdat6);

**Supervised Machine Learing with TreeBagger**

% Classification using TreeBagger

% Steps

% 1) Extract individual paramter data from each peak

% 2) concatenate data into the appropriate array arrangement with 'feature' as headings

% 3) Create/train an algorithm using TreeBagger

% 4) Measure accuracy (confusion matrix)

% 5) Additional measure of accuracy (Performance curve)

% 6) Simplify model (determine feature importance, see classifcation video for TreeBagger output syntax--already calculated by TreeBagger))

% 7) Rerun training by running TreeBagger again with indexed (limited to important features) feature matrix

% 8) Plot classification errors with all variables vs indexed variables

% 9) Use classification model on blinded data to assess predicted outcome

% Train a Bagged Ensemble of Classification Trees

close all

clear all

clc

% randomize

% extract 33% of a given file

% concatenate 33% into learning dataset

% concatenate remaining 66% into test dataset

% build both data sets simultaneously

% run learning on 33%

% validate on 66%

% export probabilities of each peak determiantion during validation

% Concatenate data

z='y';

while z=='y'

filename = uigetfile;

open(filename);

C=ans.ds;

learn=randperm(length(C),round(.33*length(C)));

for i=1:length(learn)

learn_ds(i,:)=C(learn(i),:);

end

B=sort(learn);

j=1;

q=1;

for k=1:length(C)

if k==B(j)

if j==length(learn)

else

j=j+1;

end

else

test_ds(q,:)=C(k,:);

q=q+1;

end

end

concat_learn = vertcat(concat_learn,learn_ds);

concat_test = vertcat(concat_test,test_ds);

filename

prompt = 'Add more data (y or n)? \n';

z = input(prompt,'s');

end

concat_learn.Properties.VarNames{5} = 'hplateau';

uisave('concat_learn','concatenate_learn_data_set');

uisave('concat_test','concatenate_test_data_set');

'Open a concatenated learn data set'

uiopen

'Open a concatenated test data set'

uiopen

% Train a bagged ensemble of classification trees using the data and specifying 50 weak learners. Store which observations are out of bag for each tree.

rng(1); % For reproducibility

features = [concat_learn.maximum concat_learn.mup concat_learn.mdown concat_learn.w concat_learn.hplateau];

NTrees = 50;

BaggedEnsemble = TreeBagger(NTrees,features,concat_learn.treatment,'oobvarimp','on','OOBPred','On');

% Plot the out-of-bag error over the number of grown classification trees.

figure

plot(oobError(BaggedEnsemble))

xlabel('Number of grown trees')

ylabel('Out-of-bag classification error')

% Measure accuracy

% Matrix

[oobPredictions, oobScores]=oobPredict (BaggedEnsemble);

[conf,classorder]=confusionmat(concat_learn.treatment,oobPredictions);

disp(dataset({conf,classorder{:}},'obsnames',classorder))

% Performance Curve

[Yfit,Sfit]=BaggedEnsemble.oobPredict;

treatment={'control';'propranolol';'isoproterenol'};

mapRatings=ordinal(treatment, [],BaggedEnsemble.ClassNames);

mapRatings=double(mapRatings);

figure

for j=1:length(treatment)

[fpr,tpr,gar,auc]=perfcurve(concat_learn.treatment,Sfit(:,mapRatings(j)),BaggedEnsemble.ClassNames{mapRatings(j)});

subplot(1,3,j);

plot(fpr,tpr)

xlabel('False Positive Rate')

ylabel('True Positive Rate')

title(BaggedEnsemble.ClassNames{j})

legend(['AUC = ' num2str(auc)],'Location','southeast')

end

% Simplify Models

% Estimate variable importance

figure

bar(BaggedEnsemble.OOBPermutedVarDeltaError);

xlabel('Treatment')

ylabel('Out-of-bag feature importance');

title('Feature Importance Results');

set(gca, 'XTick',[1,2,3,4,5], 'XTickLabel',concat_learn.Properties.VarNames)

% Save compact version of TreeBagger

c=compact(BaggedEnsemble);

oobErrorFullX=BaggedEnsemble.oobError;

% Reduce Features Based on Results of Above Estimate

features = [concat_learn.maximum concat_learn.mup concat_learn.mdown concat_learn.w concat_learn.hplateau];

NTrees = 50;

BaggedEnsemble2 = TreeBagger(NTrees,features(:,[1 4 5]),concat_learn.treatment,'oobvarimp','on','OOBPred','On');

%Further Reduced Features Down to Two

BaggedEnsemble3 = TreeBagger(NTrees,features(:,[1 5]),concat_learn.treatment,'oobvarimp','on','OOBPred','On');

oobErrorReduced=BaggedEnsemble2.oobError;

oobErrorTwoPar=BaggedEnsemble3.oobError;

figure

plot(oobErrorFullX,'b')

hold on

plot(oobErrorReduced, 'r')

hold on

plot(oobErrorTwoPar, 'g')

xlabel('Number of grown trees');

ylabel('Out-of-bag classification error');

legend({'All features', 'Features 1,4,5', 'Features 1,5'},'Location','NorthEast');

title('Classification Error for Different Sets of Predictors');

hold off;

% Measure accuracy

% Matrix

[oobPredictions2, oobScores2]=oobPredict (BaggedEnsemble2);

[oobPredictions3, oobScores3]=oobPredict (BaggedEnsemble3);

[conf,classorder]=confusionmat(concat_learn.treatment,oobPredictions2);

[conf3,classorder3]=confusionmat(concat_learn.treatment,oobPredictions3);

disp(dataset({conf,classorder{:}},'obsnames',classorder))

disp(dataset({conf3,classorder3{:}},'obsnames',classorder3))

% Performance Curve

[Yfit,Sfit2]=BaggedEnsemble2.oobPredict;

treatment={'control';'propranolol';'isoproterenol'};

mapRatings=ordinal(treatment, [],BaggedEnsemble2.ClassNames);

mapRatings=double(mapRatings);

%Performance Curve 2

[Yfit,Sfit3]=BaggedEnsemble3.oobPredict;

treatment={'control';'propranolol';'isoproterenol'};

mapRatings=ordinal(treatment, [],BaggedEnsemble3.ClassNames);

mapRatings=double(mapRatings);

figure

title('Super Reduced Model')

for j=1:length(treatment)

[fpr,tpr,gar,auc]=perfcurve(concat_learn.treatment,Sfit(:,mapRatings(j)),BaggedEnsemble.ClassNames{mapRatings(j)});

[fpr2,tpr2,gar2,auc2]=perfcurve(concat_learn.treatment,Sfit2(:,mapRatings(j)),BaggedEnsemble2.ClassNames{mapRatings(j)});

[fpr3,tpr3,gar3,auc3]=perfcurve(concat_learn.treatment,Sfit3(:,mapRatings(j)),BaggedEnsemble3.ClassNames{mapRatings(j)});

subplot(1,3,j);

plot(fpr,tpr,'b')

hold on

plot(fpr2,tpr2,'r')

hold on

plot(fpr3,tpr3, 'g')

xlabel('False Positive Rate')

ylabel('True Positive Rate')

title(BaggedEnsemble2.ClassNames{j})

legend(['AUC5 = ' num2str(auc)], ['AUC3 = ' num2str(auc2)], ['AUC2 = ' num2str(auc3)], 'Location','southeast')

end

% Estimate variable importance

figure

bar(BaggedEnsemble2.OOBPermutedVarDeltaError);

xlabel('Treatment')

ylabel('Out-of-bag feature importance');

title('Feature Importance Results');

namesmatrix145= [concat_learn.Properties.VarNames(1),concat_learn.Properties.VarNames(4),concat_learn.Properties.VarNames(5)];

set(gca, 'XTick',[1,2,3,4,5], 'XTickLabel',namesmatrix145)

% Estimate variable importance 3

figure

bar(BaggedEnsemble3.OOBPermutedVarDeltaError);

xlabel('Treatment')

ylabel('Out-of-bag feature importance');

title('Feature Importance Results');

namesmatrix15= [concat_learn.Properties.VarNames(1),concat_learn.Properties.VarNames(5)];

set(gca, 'XTick',[1,2,3,4,5], 'XTickLabel',namesmatrix15)

% Save compact version of TreeBagger

c=compact(BaggedEnsemble);

d=compact(BaggedEnsemble2);

e=compact(BaggedEnsemble3);

% Predict unknown data without classifiers and compute probability of each

% classification

concat_test=double(concat_test(:,1:5));

concat_test2=double(concat_test(:,[1 4 5]));

concat_test3=double(concat_test(:,[1 5]));

[YFIT,SCORES] = c.predict(concat_test);

prediction=dataset(YFIT,SCORES);

[YFIT2,SCORES2] = d.predict(concat_test2);

prediction2=dataset(YFIT2,SCORES2);

[YFIT3,SCORES3] = e.predict(concat_test3);

prediction3=dataset(YFIT3,SCORES3);
